# Species-specific rumen microbial responses to dietary inhibition of methanogenesis in cows and goats

**DOI:** 10.3389/fmicb.2026.1852812

**Published:** 2026-09-04

**Authors:** Karla Fabiola Corral-Jara, Yuliaxis Ramayo-Caldas, Laurence Bernard, Cécile Martin, Jeremy Tournayre, Diego P. Morgavi, Milka Popova

**Affiliations:** 1 Université Clermont Auvergne, INRAE, VetAgro Sup, UMR 1213 Herbivores Unit, Saint-Gènes-Champanelle, France; 2 Animal Breeding and Genetics Program, IRTA, Caldes de Montbui, Spain; 3 Université Paris-Saclay, INRAE, AgroParisTech, GABI, Jouy-en-Josas, France

**Keywords:** cows, goats, diet, methane, rumen, metatranscriptomic

## Abstract

**Introduction:**

Methane, a greenhouse gas, is produced in the rumen microbiome of ruminants. Various nutritional strategies can reduce enteric methane (CH_4_) emissions from livestock, but it is unclear whether diet affects rumen microbiome similarly across ruminant species. The objective of this study was to determine whether cows and goats differ in their rumen microbial functional responses to dietary strategies based on starch and/or lipid supplementation, and whether these differences explain diet-associated variation in CH_4_ emissions under comparable experimental conditions.

**Methods:**

Experiments were conducted simultaneously, and both species received the same diet based on grassland hay and concentrate as the Control diet (CTL) or supplemented with corn oil and wheat starch (COS), marine algae powder (MAP) or hydrogenated palm oil (HPO). To identify biologically relevant features from the rumen microbial metatranscriptomes, we followed a five-step integrative statistical and network analysis pipeline, combining a network-based approach with clustering and supervised model fitting to associate differentially expressed genes and taxa with CH_4_ emissions in the rumen.

**Results:**

The COS diet lowered CH_4_ emissions in both cows and goats and altered their rumen microbiomes. However, the number of differentially expressed KEGG orthologs and differentially abundant OTUs identified in the COS vs CTL comparison was greater in cows than in goats. Moreover, clustering analysis revealed differences in network topology between ruminant species. In goats, CH_4_ emission reduction was strongly associated with genes involved in carbohydrate metabolism and methylotrophic and hydrogenotrophic pathways of methanogenesis; whereas in cows, hydrogenotrophic pathways were prominent. Additionally, sparse Partial Least Squares (sPLS) analysis identified species-specific discriminant microbial features.

**Discussion:**

Overall, these results show that host species modulates rumen microbial responses to diet and suggest that microbial interactions underlying CH₄ mitigation differ between cows and goats, although validation in independent studies involving larger sample sizes is required.

## 1 Introduction

Ruminants have the unique ability to transform plant biomass into nutrient-dense foods because of a specialized microbiome that resides in the rumen digestive segment. However, ruminant production is under strong societal pressure due to its high carbon footprint associated with methane (CH_4_) emission, a potent greenhouse gas (GHG) (McMichael et al., 2007). In milk production systems, cows are the most important source of enteric CH_4_, but dairy goats, sheep, and buffalo also contribute (Gerber et al., 2013). This trend is consistent with previous summaries of global dairy goat production reporting a marked increase in goat milk production over recent decades, including a 62% increase between 1993 and 2013 (Miller and Lu, 2019). In addition, goats are particularly suited to harsh climatic conditions (Pragna et al., 2018) and linked to climate changes, their population and production areas are likely to expand, replacing other livestock species.

Methane production in ruminants is driven by methanogenic archaea that live in the rumen (Balch et al., 1979). These microorganisms rely on products generated during the fermentation of feed by bacteria and other microbes under anaerobic conditions. During this process, complex dietary components such as plant fibers are broken down into simpler compounds, releasing gasses like hydrogen (H₂) and carbon dioxide (CO₂). Methanogens primarily use H₂ to convert CO₂ into methane (CH₄), helping to maintain efficient fermentation by removing excess hydrogen. In addition to this main pathway (hydrogenotrophy), some methanogens can also produce methane through methylotrophy, using methyl-containing compounds such as methanol or methylamines as alternative substrates (Morgavi et al., 2010).

Nutritional strategies appear to be an effective way to mitigate ruminant CH_4_ emissions (Huws et al., 2018) that farmers could quickly adopt without altering the efficiency of animals. Several studies comparing high- and low-CH₄-emitting animals or diets have reported associations between CH₄ emissions and ruminal microbial taxa, metagenomic gene abundance, or microbial interaction networks. For example, Auffret et al. (2017) identified microbial biomarkers associated with methane emissions across cattle breeds and basal diets, whereas Roehe et al. (2016) and Wallace et al. (2015) highlighted the contribution of metagenomic gene abundance to methane-emission phenotypes. More recently, it was suggested that variation in methane emissions may be driven by complex interactions among microbial groups occupying different functional niches (Martínez-Álvaro et al., 2020; Ramayo-Caldas et al., 2020). Thus, although previous studies support a microbial basis for variation in CH₄ emissions, they do not point to a single, universal microbial marker or mechanism. These differences may reflect variation in host background, basal diet, mitigation strategy, sampling time, sequencing approach, and statistical framework, and highlight the need for integrative analyses linking CH₄ phenotypes with microbial taxonomic and functional activity. Moreover, most studies have focused on a single ruminant species, predominantly cattle (Ortiz-Chura et al., 2024), limiting our understanding of whether microbial signatures associated with CH₄ emissions are conserved across species or shaped by species-specific differences in rumen physiology, feeding behavior, and digesta turnover. Comparative studies across ruminant species are therefore needed to determine the extent to which microbial responses to CH₄-mitigating diets are shared or host-dependent.

A previous study found that diet supplemented with corn oil and wheat starch reduced CH₄ emissions in both dairy cows and goats, whereas diets supplemented with marine algae powder or hydrogenated palm oil had no significant effect on CH₄ emissions (Martin et al., 2021). This design provided a unique opportunity to investigate whether cows and goats differ in the rumen microbial mechanisms associated with diet-induced variation in CH₄ emissions. Here, we used rumen metatranscriptomic data from cows and goats fed the same control diet and three starch- and/or lipid-supplemented diets under comparable management conditions. The primary objective was to determine whether host species shapes the microbial functional response to dietary strategies affecting CH₄ emissions. We hypothesized that, because of species-specific rumen conditions, cows and goats would differ in the microbial interactions and methanogenesis-related pathways associated with diet-induced variation in CH₄ emissions. To test this hypothesis, we integrated metatranscriptomic profiles with CH₄ emission traits and rumen fermentation parameters using differential expression analysis, co-occurrence networks, pathway enrichment, and sparse Partial Least Squares analyses (sPLS).

## 2 Methods

### 2.1 Experimental methods

The Auvergne Rhône-Alpes Ethics Committee approved the experimental protocol for Animal Experiments (France; DGRI agreement APAFIS#3277–2015121411432527 v5), which conformed with European Union Directive 2010/63/EU norms. Experiments were carried out at the U. E. Herbipôle Low Mountain Ruminant Farming Systems Facility, https://doi.org/10.15454/1.5572318050509348E12 at Theix, 63122 Saint Genès Champanelle from February to July 2016. Trials with cows and goats were conducted simultaneously; the experimental approach is depicted in Supplementary Figure 1A.

### 2.2 Diets, animals, and experimental design

Four lactating multiparous Holstein cows and four Alpine goats were used in two parallel 4 × 4 Latin-square designs, one for each species. Each animal received each of the four dietary treatments across four experimental periods (Fougère et al., 2018; Martin et al., 2021), resulting in four observations per diet within each species and 32 rumen samples in total for metatranscriptomic analysis. Briefly, animals were randomly assigned to one of four experimental diets: (i) CTL grassland hay and concentrate in a 45:55 ratio on a dry matter (DM) basis containing no additional lipid, (ii) COS, which was a CTL diet supplemented with corn oil (5.0% of total dry matter intake (DMI)) (Olvea, Saint Léonard, France) and wheat starch, (iii) MAP, CTL + 1.5% DMI of *Schizochytrium* sp. marine algae powder (DSM, Basel, Switzerland), or (iv) HPO, CTL + 3% DMI of hydrogenated palm oil (Provimi, Cargill, Saint-Germain-en-Laye, France). Dietary formulations are detailed in previous works (Fougère et al., 2018; Martin et al., 2021) and summarized in Supplementary Table 1. Each experimental period lasted 28 days, and at the beginning of each period, animals were adapted to the diet for 6 days. Experimental diets were fed ad libitum twice daily (08 h30 and 16 h00) except from day 21 to 27 when animals were moved to open-circuit respiration chambers and received 95% of individual voluntary feed intake to ensure total feed consumption. The animals had access to a constant supply of fresh water ad libitum.

### 2.3 Measurements, sampling, and chemical analysis

#### 2.3.1 Enteric CH_4_ emissions

Enteric CH₄ emissions were measured in open-circuit respiration chambers from day 21 to day 27 of each experimental period, as previously described (Martin et al., 2021). Daily CH₄ production was expressed as g/day. CH₄ yield was calculated per unit of dry matter intake (g/kg DMI), and CH₄ intensity was calculated per unit of milk yield (g/kg milk) or fat- and protein-corrected milk yield (g/kg FPCM). Individual CH₄ production, CH₄ yield, CH₄ intensity, and CH₄/CO₂ ratio were used as methane-emission traits in the integrative analyses.

#### 2.3.2 Ruminal fermentation parameters

Ruminal fluid samples (500 mL) were collected by stomach tubing (Martin et al., 2021) before the morning feeding on day 27 of each experimental period after the respiration-chamber measurement period. The first fraction was discarded to minimize saliva contamination, and ruminal fluid was homogenized before subsampling. Subsamples were collected for volatile fatty acid (VFA), ammonia (NH₃), protozoa, and RNA analyses. VFA and NH₃ concentrations were determined as previously described and expressed as mmol/L (Morgavi et al., 2008). Protozoa were fixed in an Methyl Green–Formalin–Saline (MFS) solution after sampling and counted by microscopy (Bougouin et al., 2018). Counts were log-transformed and classified as small *Entodiniomorphs* (<100 μm) (small_Ento) or large *Entodiniomorphs* (>100 μm) (large_Ento) or as *Holotrichs* [*Dasytricha (Dasy)* or *Isotricha (Iso)*] (Williams and Coleman, 1997). For RNA extraction, 15 mL of ruminal fluid was collected immediately after homogenization, snap-frozen in liquid nitrogen, and stored at −80 °C until extraction (Popova et al., 2017).

#### 2.3.3 Rumen microbial RNA extraction and sequencing

Ruminal samples were ground under liquid nitrogen and RNA was extracted from frozen rumen material using the RNeasy Plus Mini Kit (Qiagen) (Popova et al., 2017). RNA concentration was measured using a NanoDrop-1000 Spectrophotometer (Thermo Fisher Scientific), and RNA integrity was assessed using a BioAnalyzer 2100 system (Agilent Technologies). RNA quantity and integrity were checked before sequencing, with approximately 3 μg of RNA submitted per sample and RNA integrity number (RIN) greater than 8. Residual DNA was removed using the Turbo DNA-free kit (Life Technologies), and RNA was further purified and concentrated using the RNA Clean and Concentrator kit (Zymo Research). Total extracted RNA was sent to Center d’expertise et de services Génome Québec for Illumina NovaSeq 4000 paired-end sequencing. Raw data are available in the link at the data and material availability section.

## 3 Bioinformatic analysis

Shotgun metatranscriptomic data from 32 rumen content samples obtained from Holstein cows and Alpine goats were analyzed using the MetaTrans open-source pipeline (Martinez et al., 2016) Raw paired-end reads were subjected to quality control using FastQC tool (Andrews, 2010) and the Kraken pipeline (Wood and Salzberg, 2014), based on per-base N content (<5%), read length (minimum 150 bp), and mean per-sequence quality score (>27). SortMeRNA (Kopylova et al., 2012) was used to separate ribosomal RNA or tRNA (rRNA/tRNA reads) from non-rRNA/tRNA reads, the latter being retained as putative mRNA reads for functional analysis. The rRNA/tRNA read fraction was used only for taxonomic profiling. Paired-end reads were first overlapped using Fastq-Join (Aronesty, 2013), clustered using UCLUST (Edgar, 2010) and grouped into operational taxonomic units (OTUs) at 99% sequence similarity. These OTUs were taxonomically annotated against the SILVA v138.1 16S rRNA gene database using SOAP2 (Li et al., 2009).

For functional annotations, the non-rRNA/tRNA reads were mapped using the SOAP2 to a bovine rumen microbial gene catalog (Li et al., 2020). Putative genes were predicted using FragGeneScan (Rho et al., 2010) and clustered using CD-HIT v4.6 (Fu et al., 2012) with an identity threshold of > = 95% and gene overlap of > = 90%. Functional annotation was performed using the Kyoto Encyclopedia of Genes and Genomes (KEGG) database and features assigned to KEGG orthologs (KOs) with >50% assignment were retained. The bioinformatic analysis metrics are shown in Supplementary Table 2.

In this study, “gene expression” refers to microbial metatranscriptomic transcript abundance and not to host gene expression. KOs were used to describe microbial functional transcript profiles derived from the non-rRNA/tRNA read fraction, whereas OTUs were used as taxonomic features derived from the rRNA/tRNA read fraction. The term “functional activity” is used here to describe the relative abundance of microbial transcripts assigned to functional categories or pathways. Features identified by sPLS analysis are referred to as candidate microbial markers or discriminant microbial features.

## 4 Statistical and network analysis

Cows and goats were analyzed as two parallel 4 × 4 Latin-square designs, with four animals per species, four experimental periods, and four dietary treatments. Animal, period, and treatment effects were considered in the analysis of methane-emission and rumen fermentation traits, as described in the previously reported animal-performance analysis (Martin et al., 2021). In the present metatranscriptomic analysis, these individual phenotypic traits were integrated with microbial taxonomic and functional profiles to explore diet-associated microbial mechanisms within each species. Because of the limited number of animals and the high dimensionality of the metatranscriptomic data, differential occurrence, network, clustering, and sPLS analyses were considered exploratory. Therefore, microbial features identified by these analyses were interpreted as candidate or discriminant features associated with diet and CH₄ emissions, rather than as validated biomarkers.

As an initial exploratory analysis, the analysis of similarities [anosim function from the *vegan* R package (Oskansen et al., 2016)], was used to test whether microbial community composition differed among experimental groups based on dissimilarity matrices. Bray–Curtis dissimilarities were calculated from OTU and KO profiles. The betadisper function was used to assess differences in dispersion among groups. Principal Coordinates Analysis (PCoA) was used to visualize variation in OTU and KO profiles.

To identify candidate microbial features and explore their relationships with methane-emission phenotypes, we implemented a five-step integrative statistical and network analysis pipeline depicted in Supplementary Figure 1B, which included the sequential steps of differential occurrence, co-occurrence networks, clustering, pathway enrichment analysis, and supervised statistical approaches such as sPLS. The pipeline was built using the R programming language. The R script and RData file are available at Code availability.

The pipeline was used to analyze pair-wise comparisons of all diets (COS vs. CTL, COS vs. MAP, COS vs. HPO, and CTL cows vs. CTL goats) and to investigate diet-dependent changes in the abundance of KEGGs and OTUs within each species.

### 4.1 Differential occurrence analysis

*DESeq2* R package (Love et al., 2014) with the Wald test were used to make differential abundance pair-wise comparisons among diets, both on transcripts and OTUs. To control the false discovery rate (FDR), the Benjamini–Hochberg (Benjamini and Hochberg, 1995) approach was used to compensate for multiple testing. KOs and OTUs with an FDR-adjusted *p* < 0.05 were considered significantly different between experimental groups. Differentially abundant KOs and OTUs are hereafter referred to as deKOs and deOTUs, respectively (Supplementary Figure 1B, step 1).

### 4.2 Co-occurrence networks analysis

In order to examine bivariate unconditional associations between variables, matrices with deKOs and deOTUs from pair-wise comparisons were used to compute co-occurrence networks using the *ccrepe* package in R (Schwager et al., 2014; Supplementary Figure 1B, step 2). The fermentation parameters total volatile fatty acids (VFAs, mmol), acetate, propionate, butyrate, isobutyrate, valerate, isovalerate, caproate represented as mmol/mmol of total VFAs, ratios acetate/propionate, acetate+butyrate/propionate, NH_3_ (mMol), DMI (kg/d), organic matter intake {OMI [(kg/d)], CH_4_ (g/d), CH_4_ yield, CH_4_/CO_2_} but also individual protozoa counts (log-transformed) (total protozoa, small_Ento, large_Ento, Iso, Dasy) were integrated into the deKOs and deOTUs input matrices.

The co-occurrence inference graph was created using Pearson correlation (Friedman and Alm, 2012). Each input matrix was divided by the sum of its corresponding column for data transformation. The Reboot approach was used to resample the edge scores. Edge scores were first randomized using 1,000 permutations of row shuffling (for null distributions), followed by 1,000 permutations of bootstrapping (for s randomization). In brief, the correlation of measure scores is used to calculate a correction factor and show the associations’ strength. A threshold of the *p*-values of the edges assigned to node pairs and the corrected significance (*q*-value) was set at *p <* 0.05 and *q*-value < 0.001. Cytoscape software (Shannon et al., 2003) was employed to visualize the inferred network and cluster.

### 4.3 Clustering analysis

A clustering algorithm was applied to categorize the co-occurrence networks into functionality (KOs) and/or taxonomically (OTUs) enriched units. We applied a K-means clustering approach using the Hartigan and Wong algorithm, implemented in the *stats* package in R (R Core Team, 2024; Supplementary Figure 1B, step 3). The matrices of significantly correlating deKOs, deOTUs and fermentation parameters were used as inputs in this analysis.

### 4.4 Pathway analysis

The *KEGGREST* package (Tenenbaum and Bioconductor Package Maintainer, 2021) was used to explore the biological relevance of network clusters. The list of deKOs of each cluster was used to find enrichment in diverse biological categories and associated KEGG pathways. KO identifiers were mapped to KEGG pathway maps using the KEGG Mapper Reconstruct Pathway tool (Kanehisa and Sato, 2020; Kanehisa et al., 2023). The deKOs of co-occurrence network clusters and their linkages with pathways and metabolic reactions were used to create a network of metabolic pathways; the pathways were then categorized by degree of interactions, and the deKOs by associated pathway (Supplementary Figure 1B, step 4).

The *KEGGREST* package was also used to investigate reactions and metabolites related to deKOs of each cluster. Cytoscape was used to visualization of results.

### 4.5 Multivariate analysis

Sparse Partial Least Square (sPLS), as implemented in the *mixomics* r package (Lê Cao et al., 2009) explore relationships between microbial cluster profiles (X matrix) and methane emission variables (Y matrices) (Supplementary Figure 1B, step 5). This projection-based method allows the analysis of associations between two continuous high-dimensional datasets. Clusters associated with the methane metabolism pathway (map00680) were related to several methane emission variables, including CH₄ production (g/day), CH₄ yield, emission intensity metrics (CH₄/milk, CH₄/FPCM), and the ratio CH₄/CO₂. Model parameters were optimized using the *perf* and *tune* functions with M-fold cross-validation (5 folds, 10 repetitions) and mean absolute error (MAE). In addition, PLS regression models were applied to each methane emission variable, and the top 25–30 features with the highest absolute loadings were extracted for interpretation.

## 5 Results

### 5.1 Metatranscriptomic profiling of rumen microbial communities in cows and goats

The experimental design underlying the integrative analyses is summarized in Supplementary Figure 1A, while the animal-level methane responses have been reported in detail by Martin et al. (2021). Briefly, the COS diet reduced CH₄ yield in both cows and goats, from 19.5 to 14.4 g/kg DMI in cows and from 19.5 to 13.5 g/kg DMI in goats. CH₄ intensity was similarly reduced, from 13.5 to 9.9 g/kg milk in cows and from 13.8 to 8.9 g/kg milk in goats. In contrast, MAP and HPO did not significantly reduce CH₄ yield or intensity compared with the control diet. The individual CH₄ emission variables were integrated with rumen fermentation parameters, microbial taxonomic profiles, and functional gene expression data to identify microbial features associated with diet-induced CH₄ mitigation (Supplementary Figure 1B).

**Figure 1:**
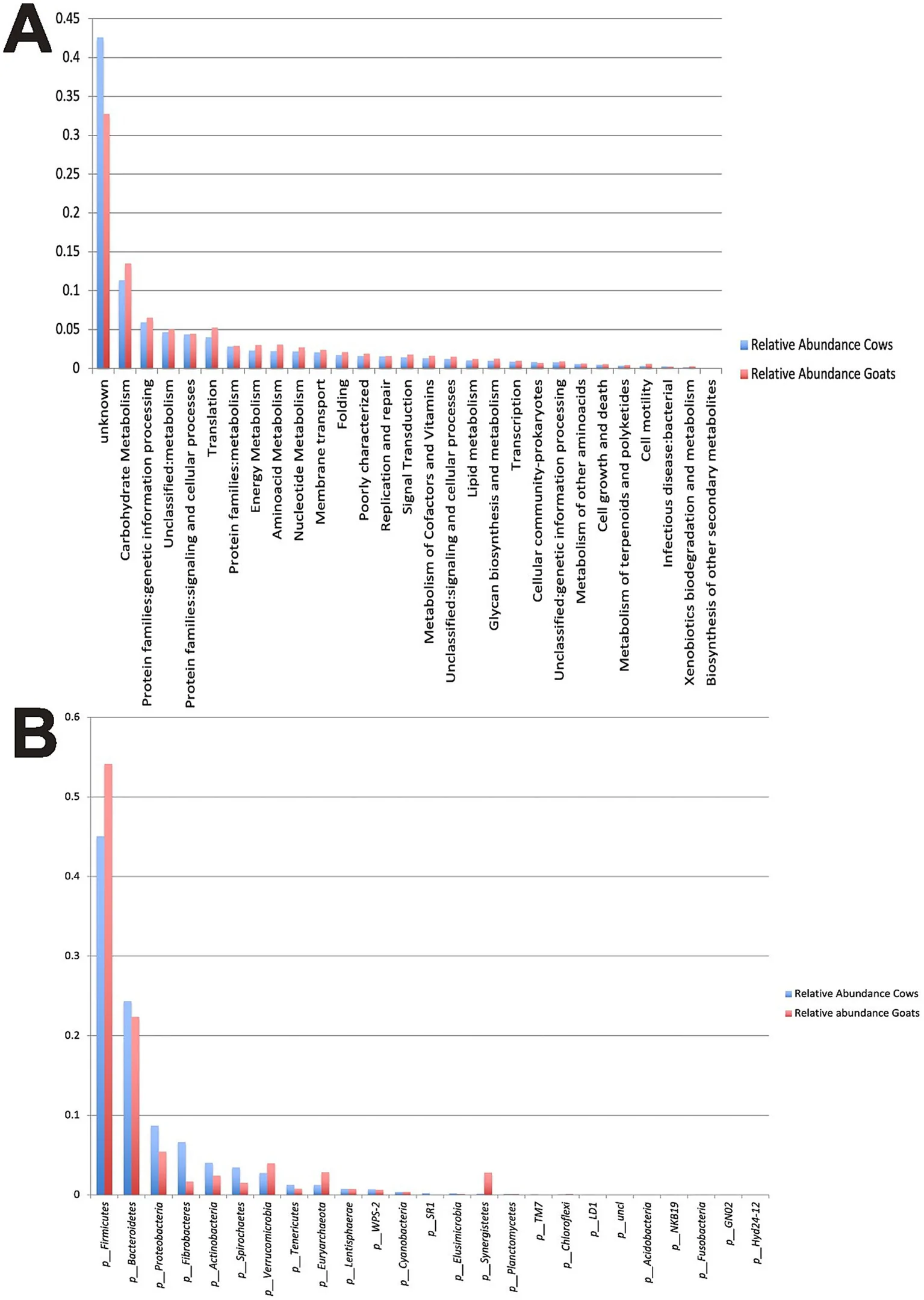
Relative abundance profiles of functional and taxonomic features in cows and goats. **(A)** Relative abundance of detected KEGG orthologs (KOs) in the quality-filtered RNA-seq dataset from cows (blue) and goats (red). **(B)** Phylum-level taxonomic composition of detected operational taxonomic units (OTUs) in the quality-filtered RNA-seq dataset from cows (blue) and goats (red).

In total, 5,132 functional genes, referred to hereafter as KEGG orthologs (KOs), and 323 OTUs were identified in cows, whereas 4,838 KOs and 246 OTUs were identified in goats. Across both species, this represented 5,235 functional genes and 368 OTUs detected in the metatranscriptomic dataset. The identified KOs were related to Carbohydrate metabolism (11 and 13% in cows and goats, respectively), Genetic information processing protein families (5 and 6.5%), Unclassified metabolism functions (4 and 5%), Signaling and cellular processes protein families (4.3 and 4.4%) and Translation (4 and 5%) (Figure 1A). Major microbial phyla were presented by Firmicutes (45% in cows and 54% in goats) and Bacteroidetes (24% in cows and 22% in goats), followed by (8% in cows and 5% in goats) (Figure 1B).

### 5.2 Comparative analysis of cows’ and goats’ microbiomes under control and supplemented diets

Microbial composition (differentially abundant OTUs, deOTUs) and functional activity (differentially abundant KEGG orthologs, deKOs) were initially examined in cows vs. goats for the CTL diet to provide a meaningful basis for analysis. At the phylum level, there was no significant difference in relative abundances, except for Fibrobacteres, Euryarchaeota and some low abundant phyla like *WPS.2, SR1,* Synergistetes and Tenericutes. We identified only 16 deOTUs significantly different between CTL cows and CTL goats in a differential analysis. Two deOTUs were classified as methanogens, *Methanobrevibacter* and *VadinCA11* (an OTU from the Methanomassiliicoccales order) and were more abundant in goats. Inversely, a deOTU affiliated with *Fibrobacter succinogenes* represented 0.3% of total OTU counts in CTL cows and less than 0.1% in goats. Similarly, two *Sharpea*-related deOTUs were more abundant in cows, while two *Oscillospiraceae* and one *Veillonellaceae* presented higher abundance in goats. The other significantly abundant deOTUs grouped less than 0.001% of the total counts.

**Figure 2:**
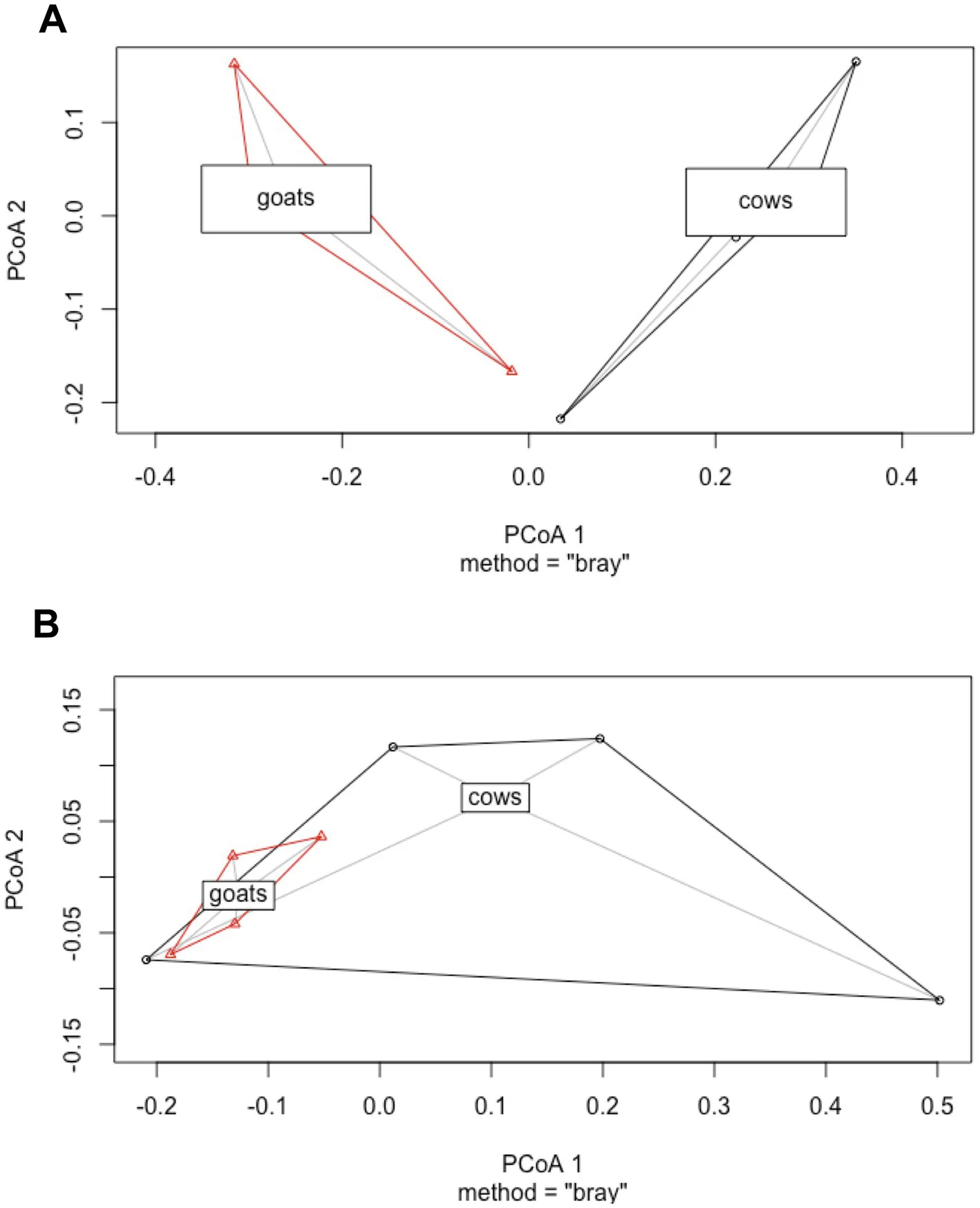
Principal Coordinates Analysis (PCoA) of rumen taxonomic and functional profiles in cows and goats receiving the control diet (CTL). Sample ordination was based on Bray–Curtis dissimilarities calculated from **(A)** operational taxonomic unit (OTU) profiles and **(B)** KEGG ortholog (KO) profiles.

However, out of the 5,235 identified KOs, we enumerated 400 that were differentially abundant between the two animal species, supplied with CTL diet. Fourteen percent of them were related to Carbohydrate metabolism, 9.7% to Signaling and cellular processes protein families, 7% to unclassified metabolism, 6% to Genetic information processing protein families and 6% to Amino acid metabolism. Among these 400 differentially abundant deKOs, only 7 in cows and 6 in goats had relative abundances higher than 0.01%. PCoA based on OTU profiles showed species-specific clustering in animals fed the CTL diet (Figure 2A), whereas no clear separation between cows and goats was observed for KEGG ortholog profiles (Figure 2B). This pattern was supported by anosim test for OTUs (*R* = 0.7917, *p* = 0.033) but not for KOs (*R* = 0.3021, *p* = 0.057); group dispersion was assessed using betadisper (*p* = 0.053). This analysis suggests that despite some differences in rumen microbial community structure, the microbial functional activity was similar between goats and cows fed the same CTL diet and under the same simultaneous experimental conditions.

The same five-step integrative statistical and network analysis pipeline was applied to all pairwise dietary comparisons (COS vs. CTL, HPO vs. CTL, MAP vs. CTL, COS vs. HPO, COS vs. MAP, and HPO vs. MAP). Differential abundance and expression analyses revealed significant changes only in comparisons involving the COS diet (i.e., COS vs. CTL, COS vs. MAP, and COS vs. HPO), consistent with methane emissions analyses, which were reduced by 28% under the COS diet in both species. Therefore, the analyses presented below focus on the COS diet relative to the other diets, particularly CTL.

In cows, individual pair-wise comparisons between COS and other diets resulted in 16–28 differentially abundant deOTUs, with 10 of them shared in all comparisons (Supplementary Figure 2A; Supplementary Table 3). These 10 deOTUs (referred to as COS-specific OTUs) represent ~3% in abundance of all OTUs identified. It is worth mentioning that *Succinivibrionaceae*, *Roseburia*, unclassified *RF32*, *Prevotella ruminicola* related OTUs were higher for COS compared to the rest of the diets, while the abundance of *VadinCA11*, *Spirochaetaceae*, unclassified *RFP12*, unclassified Rickettsiales and Endomicrobia OTU was reduced. The pair-wise comparisons between COS and the other experimental diets identified 380–791 deKOs (Supplementary Figure 2B). For each deKO identified across all dietary comparisons, fold change variations in gene expression were consistently in the same direction (either up- or downregulated). There were 255 shared deKOs between dietary comparisons, referred to hereafter as COS-specific, and they were related to Energy Metabolism (12.5%), Carbohydrate metabolism (12.5%), Genetic information processing protein families (9.8%) and Translation (9%) (Supplementary Table 4).

In goats, the number of deOTUs and deKOs shared across the three pair-wise diet comparisons was lower than for cows. There were four COS-specific deOTUs (*R4.45B*, *uncl_Rickettsiales*, *SHD.231*, and *Paludibacter* representing 0.61% of all OTUs identified) (Supplementary Figure 2C; Supplementary Table 5) and 22 COS-specific deKOs (Supplementary Figure 2D; Supplementary Table 6). Changes in the abundance of these 22 deKOs in the different diet comparisons were always in the same direction as observed for cows. These 22 deKOs were related to Unclassified Metabolism (27%), Carbohydrate metabolism (22%), Metabolism protein families (13%), Amino acid metabolism (9%) and Energy metabolism (9%) functions.

**Figure 3:**
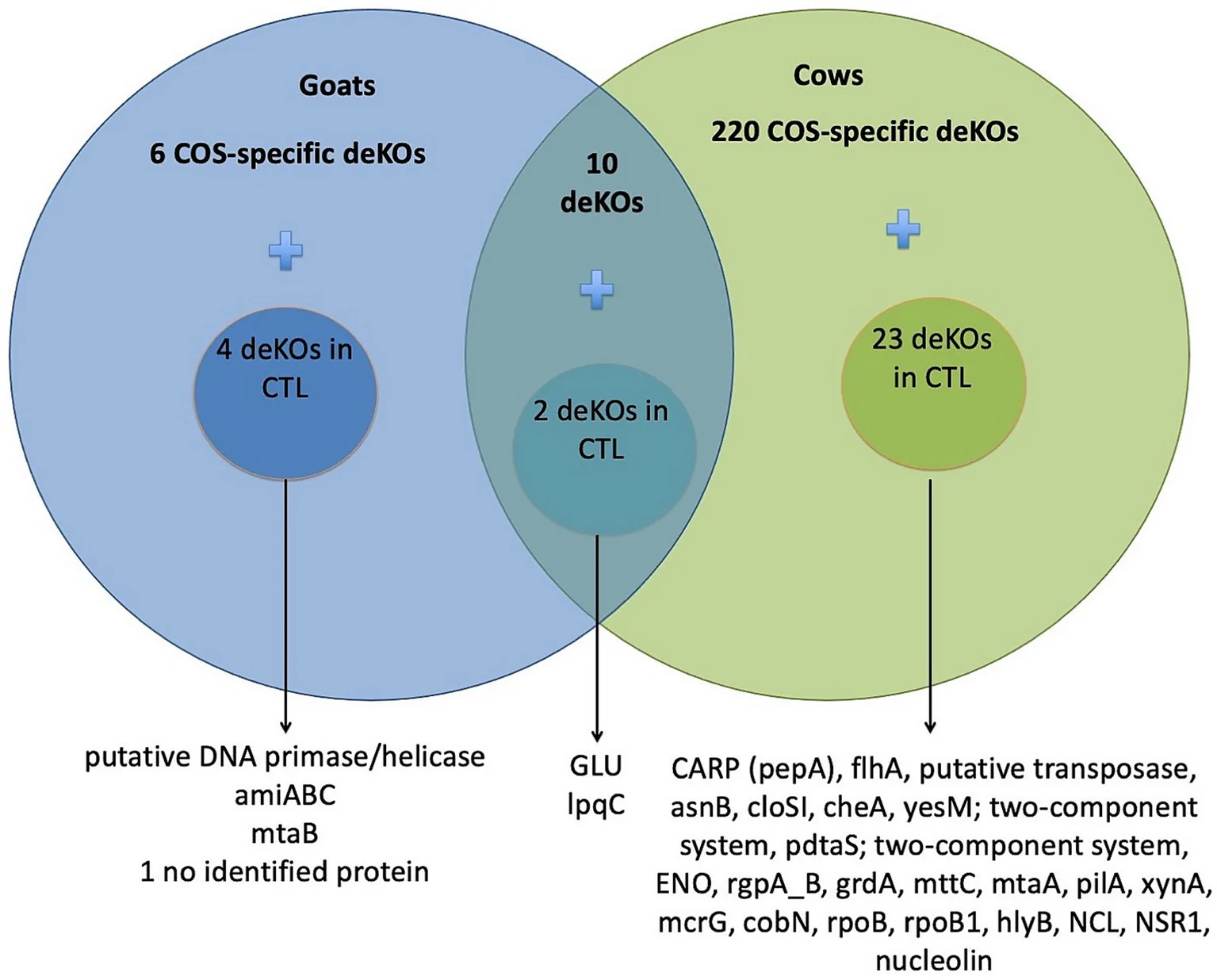
Comparison of COS-responsive differentially expressed KEGG orthologs (deKOs) between cows and goats. The Venn diagram shows KEGG orthologs that were differentially expressed between the corn oil and wheat starch supplementation treatment (COS) and the control diet (CTL) in cows and goats. The non-overlapping regions represent species-specific responses to COS, whereas the overlapping region represents deKOs shared between species. The inner circles indicate COS-responsive deKOs that also differed between cows and goats under the CTL diet. Functional annotations of these deKOs are shown below the corresponding regions.

Twenty-five of the 255 COS-specific deKOs identified in cows and six of the 22 COS-specific deKOs in goats also differed in abundance between species under CTL diets. However, most were present at low abundance (<0.01%), suggesting high similarity in gene expression between CTL-fed cows and goats (Figure 3).

For each ruminant species, co-occurrence networks were created to analyze the correlation between deKOs, deOTUs and other parameters, including VFA, CH_4_, rumen protozoa counts and intake. After clustering using the K-means function, clusters grouping CH_4_ metabolism-related variables were used in a pathway enrichment analysis.

Eleven clusters were created in the networks for all dietary comparisons in cows. The two largest clusters in the network for each dietary comparison included deKOs related to energy and carbohydrate metabolism, fermentation parameters, and CH_4_-related variables. It was observed that fermentation parameters and CH_4_ yield were always kept apart from daily CH_4_ production (CH_4_ g/d). Between dietary comparisons, over one-third of the deKOs clustering with CH_4_ variables were shared (75% in COS vs. CTL, 36% in COS vs. MAP, and 37% in COS vs. HPO).

**Figure 4:**
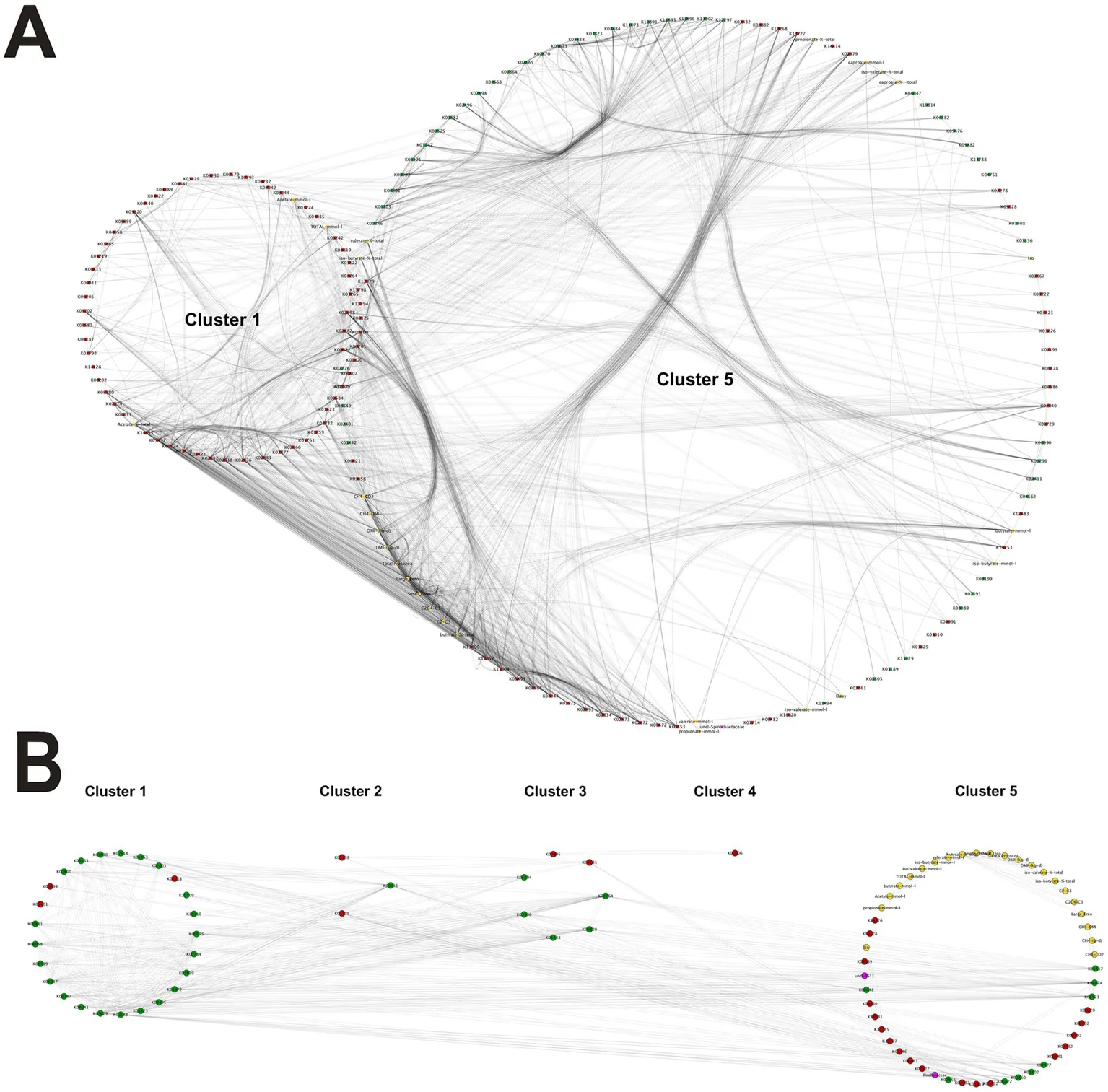
Co-occurrence networks of microbial features and rumen fermentation parameters associated with corn oil and wheat starch supplementation. Networks comprise differentially abundant operational taxonomic units (deOTUs), differentially expressed KEGG orthologs (deKOs), and fermentation parameters identified in the comparison of the COS and CTL diets in **(A)** cows and **(B)** goats. Red and green nodes represent deKOs with negative and positive log₂ fold changes, respectively, whereas pink and blue nodes represent deOTUs with negative and positive log₂ fold changes, respectively. Yellow nodes represent fermentation parameters. Network clusters are indicated by their corresponding cluster numbers.

Specifically, the COS vs. CTL network shown in Figure 4A comprised 10,902 edges, 4 of OTUs with positive fold changes (including *Succinivibrionaceae*, unclassified *RF32*, and *Prevotella ruminicola*), and 2 of the deOTUs with negative fold changes (including *Spirochaetaceae* and unclassified *RFP12*). In addition, 125 of deKOs showed positive fold changes and 159 showed negative fold changes, of which 206 were COS-specific. The network also included 28 parameters associated with VFA profiles and protozoa counts, among others (Supplementary Table 7 for COS vs. CTL, Supplementary Table 8 for COS vs. MAP and Supplementary Table 9 for COS vs. HPO).

In the COS versus CTL network of cows, clusters 1 and 5 were identified as most closely associated with the methanogenesis pathway. Cluster 5 contained CH_4_ yield, most fermentation variables, protozoa counts, an OTU classified as unclassified *Spirochaetaceae*, and 95 deKOs, primarily related to general metabolic pathways and microbial metabolism in diverse environments. Three percent of these KEGG orthologs were directly linked to the CH_4_ metabolism pathway (Supplementary Figure 3A). By contrast, cluster 1 exhibited a higher proportion of methanogenesis-related deKOs (16%), following Metabolic pathways (26%) and Microbial metabolism (20%) (Supplementary Figure 3B). These included subunits of key enzymes such as methyl-coenzyme M reductase, tetrahydromethanopterin S-methyltransferase, and formylmethanofuran dehydrogenase.

The deKOs from clusters 1, 5, and 7 (clusters containing CH_4_-related variables) and their associations with pathways and metabolic reactions were used to construct a metabolic network in cows (Supplementary Figure 4). Several pathways exhibited extensive interactions with the deKO networks, including Methane metabolism, Secondary metabolite biosynthesis, Carbon metabolism, Microbial metabolism, and General metabolic pathways. DeKOs from cluster 1 were mainly associated with the most highly connected pathways in the network, with the Methane metabolism pathway being a prominent example.

In cows, COS vs. MAP (Supplementary Figure 5) and COS vs. HPO (Supplementary Figure 6) network cluster distributions were comparable to COS vs. CTL, although *Methanosphaera* was in one of the biggest clusters (cluster 5) with CH_4_ yield and *Methanobrevibacter* clustered with CH_4_ g/d (cluster 6) in COS vs. HPO network.

The same analytical approach in COS vs. CTL of goats allowed the construction of a co-occurrence network of 518 edges containing 22 fermentation parameters, two lower abundant deOTUs (uncl_BS11 and *Pirellulaceae*), 26 higher abundant deKOs, and 37 lower abundant deKOs (Figure 4B; Supplementary Table 10). However, the co-occurrence networks for COS vs. MAP and COS vs. HPO in goats included only a few microbial variables and were not subjected to further analysis.

Clustering of the COS vs. CTL network in goats resulted in 5 clusters, two of them (cluster 1 and cluster 5) related to CH_4_ metabolism. It was noted that daily CH_4_ production was segregated in the same cluster with CH_4_ yield and fermentation parameters (cluster 5). Supplementary Figure 3C shows the pathway analysis of cluster 5, which revealed that Metabolic pathways, Microbial metabolism in diverse environments, Methane metabolism (8 deKOs), and Carbon metabolism accounted for 85% of deKOs whereas Signal transduction and Translation were represented to a lesser extent. The pathway analysis of cluster 1 from goats revealed similar pathways to those found in cluster 5, as well as Glycolysis/Gluconeogenesis, ABC transporter, Fructose and Mannose metabolism, and Glutathione metabolism (Supplementary Figure 3D).

Pathways with a high degree (more interactions in the network) were also found in the goat integration network (Supplementary Figure 7), such as Metabolic pathways, Microbial metabolism, Carbon metabolism, and Methane metabolism, which were mainly associated with a group of deKOs belonging to cluster 1 and 5 of the co-occurrence network. In addition, Sulfur metabolism, Purine metabolism, Galactose metabolism, Porphyrin metabolism, and Pentose phosphate pathways were linked with Glycolysis/Gluconeogenesis and connected with deKOs from cluster 1.

### 5.3 Candidate microbial markers associated with CH₄ emissions were identified in cows and goats

**Figure 5:**
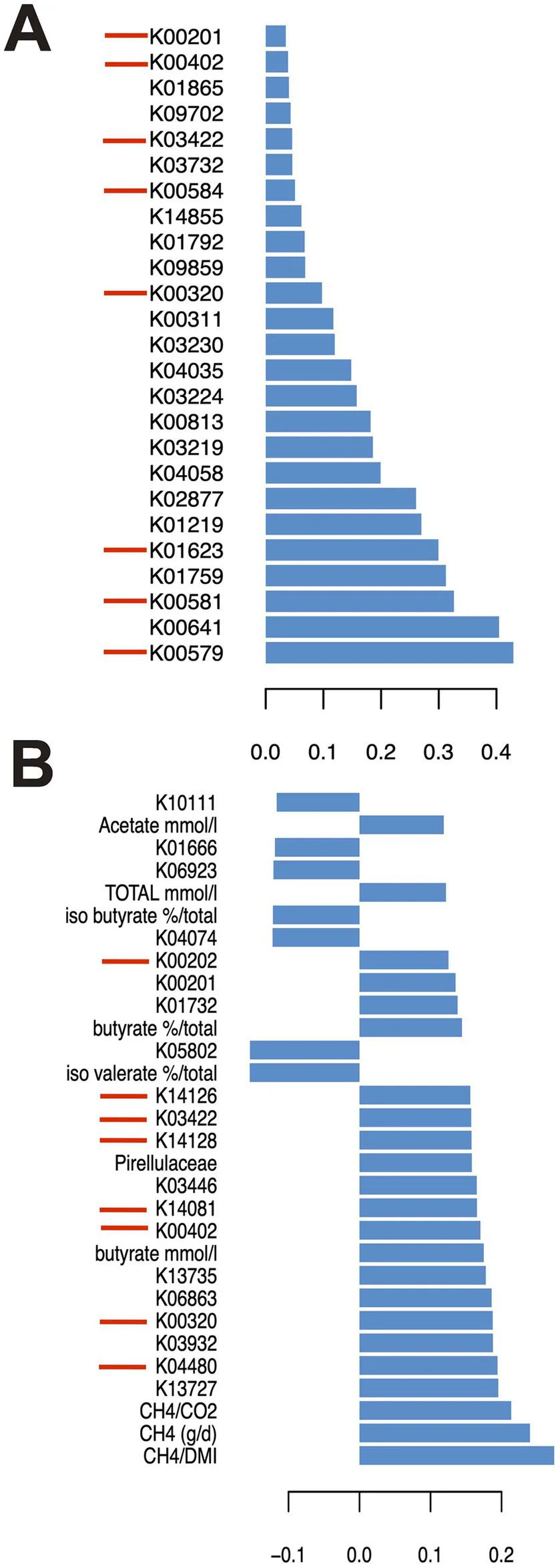
Loading plots for component 1 of the sparse partial least squares (sPLS) analyses relating microbial and fermentation profiles to methane emission variables under the COS and CTL diets in **(A)** cows and **(B)** goats. The cow analysis included 25 variables from cluster 1 of the co-occurrence network, whereas the goat analysis included variables from cluster 5. Red lines indicate KEGG orthologs assigned to the methane metabolism pathway.

To identify variables associated with CH₄ emissions within the COS vs. CTL comparison, a final sPLS regression analysis was performed using the previously selected network clusters. In cows, 25 variables from cluster 1 were retained, including eight deKOs assigned to the methane metabolism pathway (Figure 5A). In goats, 30 variables from cluster 5 were retained, including eight deKOs assigned to the methane metabolism pathway (Figure 5B). Only three discriminant deKOs were shared between the two animal species (Table 1). Moreover, all discriminant deKOs in cows and most of them in goats were less abundant in differential occurrence analysis and with positive loadings in the sPLS analysis with the COS diet, corresponding to a negative relationship with CH_4_ emission (which decreased when COS was fed to animals).

**Table 1:** Summary results of the pipeline with selected deKOs of methanogenesis as markers in goats and cows.

Cluster 1 in cows	Cluster 5 in goats	Cluster 1 in goats
K00320 (5,10-methylenetetrahydromethanopterin reductase)	K00202 (formylmethanofuran dehydrogenase subunit C)	K04041 (fructose-1,6-bisphosphatase III)
K00579 (tetrahydromethanopterin S-methyltransferase subunit C)	K00320 (5,10-methylenetetrahydromethanopterin reductase)	K00399 (methyl-coenzyme M reductase alpha subunit)
K00581 (tetrahydromethanopterin S-methyltransferase subunit E)	K04480 (methanol---5-hydroxybenzimidazolylcobamide Co-methyltransferase)	K00401 (methyl-coenzyme M reductase beta subunit)
K00584 (tetrahydromethanopterin S-methyltransferase subunit H)	K14081 (methanol corrinoid protein)	
K01623 (fructose-bisphosphate aldolase, class I)	K03422 (methyl-coenzyme M reductase subunit D)	
K03422 (methyl-coenzyme M reductase subunit D)	K00402 (methyl-coenzyme M reductase gamma subunit)	
K00201 (formylmethanofuran dehydrogenase subunit B)	K14126 (F420-non-reducing hydrogenase large subunit)	
K00402 (methyl-coenzyme M reductase gamma subunit)	K14128 (F420-non-reducing hydrogenase small subunit)	

In cows, the sPLS analysis to forecast CH_4_ (g/d) variation in each cluster showed that cluster 1 had the greatest prediction ability (58.7%), and clusters 5, 7, and 9 explained 45% of the variability. In goats, cluster 1 explained the variability of CH_4_ (g/d) by 84%, while cluster 5 by 40%. Supplementary Table 11 presents the detailed results of the analysis, with sheets A–B corresponding to cows and sheets C–D corresponding to goats.

## 6 Discussion

Rumen microorganisms play a fundamental role in feed degradation through their growth, metabolic activity, and interactions within the rumen ecosystem and with the host animal (Morgavi et al., 2015). Enteric CH₄ production is largely determined by the balance between hydrogen-generating and hydrogen-utilizing pathways during microbial carbohydrate fermentation (Ungerfeld, 2020). Recent studies suggest that a deeper understanding of the microbial mechanisms governing this balance is essential for predicting variations in methane emissions and for developing more effective mitigation strategies (Morgavi et al., 2023; Roques et al., 2024). Diet and feed additives are the most effective strategies to reduce enteric microbial CH_4_ emissions in ruminants; but while these strategies were extensively tested in cows, data on goats are scarce and inconsistent (Hatew et al., 2015; Guyader et al., 2016; Zhang et al., 2019; Marcos et al., 2020). Our previous paper reported that the COS diet, supplemented with corn oil and wheat starch, reduced CH_4_ yield relative to the CTL diet to the same extent in cows and goats, whereas the HPO and MAP diets, supplemented with hydrogenated palm oil and seaweed powder, respectively, had no effect on CH_4_ emissions (Martin et al., 2021).

The similar reduction in CH₄ emissions observed in cows and goats fed the COS diet raised the question of whether comparable phenotypic outcomes were supported by similar or distinct microbial responses. Our results suggest that this was only partly the case: although cows and goats showed comparable CH₄ yields under the control diet and similar CH₄ mitigation under COS, the microbial features associated with these responses differed between species.

Although diet is considered the primary driver of rumen microbial composition, species-associated differences in the abundance of specific microbial taxa have been reported across ruminants (Huws et al., 2018). In a large-scale survey, Henderson et al. (2015) identified Veillonellaceae and *Fibrobacter* as key taxa contributing to the distinction between bovine and caprine rumen microbiomes. Consistent with these observations, we also observed a higher abundance of *Fibrobacter* and a lower abundance of *Veillonellaceae* in cows compared to goats when receiving the CTL diet. Although cows and goats differed in microbial community structure under the CTL diet, we found no evidence of major differences in KOs between species. This limited functional divergence is consistent with the comparable CH₄ yields observed in cows and goats fed the CTL diet, suggesting that different microbial community structures may support broadly similar fermentation and methanogenesis functions.

In contrast, changes in CH_4_ emissions due to diet were better correlated with gene expression than with microbial taxonomy, as the number of deKOs was higher than that of deOTUs across diets in both species. A change in the rumen microbiota composition could have been expected as a consequence of the lipids supplemented in the diet; toxic effects were previously reported in pure cultures (Henderson, 1973) and *in vitro* (Vargas et al., 2020) and, to some extent, *in vivo* (Pitta et al., 2018). However, there was no change in microbial gene expression when cows and goats received the HPO and MAP diets compared to the control. As discussed in our companion paper (Martin et al., 2021) the lipids supplementation level applied with these diets was low (up to 3% of DMI), and supplementing fats up to 6% had shown no adverse effects on total nutrient digestibility and total VFAs (Patra, 2013) with probably limited detrimental effects on rumen microbes. The COS diet was the only one that affected microbial gene expression, demonstrating a shift in microbial metabolism. In this case, the diet’s effect can be attributed to the association of high starch and lipid supply.

The COS diet considerably modified microbial gene expression in cows and to a much lower extent in goats, as attested by the number of deKOs shared between all dietary comparisons and identified for each species, i.e., 255 for cows and 22 for goats. Nevertheless, in both species, carbohydrate metabolism was one of the primary categories affected by the diet indicating a switch in fermentation pathways.

Correlations between deKOs, deOTUs, and fermentation parameters, including individual CH_4_ measures and protozoa counts, were used to construct a compositionally-corrected network; this step, combined with the k-means clustering, assured the selection of biologically relevant microbial correlation data. A network was depicted for each dietary comparison in cows, while for goats, we used only the COS vs. CTL network (although smaller than those in cows) as COS vs. MAP and COS vs. HPO comparisons resulted in insignificant correlations between variables. The disparity between cows and goats here could be partly related to the time of sampling and to species-specific feeding behavior and rumen dynamics. Rumen content samples were taken before the morning feeding when rapidly fermentable organic matter was likely less available; in this context, identified deKOS may reflect sustained and long-lasting changes in microbial gene expression. In addition, as reported in the companion study (Martin et al., 2021) goats ate faster and more frequently and had a higher ruminal turnover rate than cows. These differences may modify substrate availability and the temporal dynamics of fermentation, thereby contributing to differences in microbial interactions and the availability of reducing equivalents for methanogenesis. However, feeding behavior and rumen turnover were not directly tested as causal drivers of the metatranscriptomic responses in the present analysis, and this interpretation should therefore be considered as a plausible biological explanation rather than a demonstrated mechanism.

A common characteristic between cows and goats is the clustering of CH_4_ yield predominantly with genes involved in various metabolic pathways and excluding most genes of the methanogenesis pathway, forming another cluster in the networks. Taking the analysis a step further, we identified the processes involved in the CH_4_ variability for each identified cluster. In goats, the variability of CH_4_ emissions was explained primarily by Carbohydrate metabolism processes. In contrast, in cows, this variability was explained by Carbohydrate and Methane metabolism clusters. These results suggest that the amount of CH_4_ produced is closely related to the activity of bacteria providing substrates for methanogenic archaea. This interpretation is also supported by a complementary metagenomic and metaproteomic analysis performed on samples from the same CTL and COS treatments (Andersen et al., 2023), which suggested that reduced CH₄ production under COS was associated with lower detectable protozoal activity and a redirection of hydrogen toward succinate- and propionate-producing bacteria rather than methanogenesis. This agrees with our metatranscriptomic results showing that CH₄-associated features involved carbohydrate metabolism and microbial interaction patterns in addition to methanogenesis-related genes.

Our analysis revealed that the enzymes tetrahydromethanopterin S-methyltransferase (Mtr) and fructose bisphosphate aldolase (Fba) characterized the relationship between cows’ microbiome and CH_4_ emission, whereas Methanol:coenzyme M methyltransferase (Mta), F420-non-reducing hydrogenase (Mvh) were highlighted in goats. The 5,10-methylenetetrahydromethanopterin reductase (Mer), methyl-coenzyme M reductase (Mcr), and formylmethanofuran dehydrogenases (Fwd) enzymes were found in both species; the expression of these genes was downregulated by COS supplementation.

Likewise, another point of difference was that the enzyme Mta, involved in the methylotrophic pathway, was specific to goats receiving the COS diet. Methylotrophs have a lower H_2_ threshold and a thermodynamic advantage in that they are driven by the availability of methyl compounds rather than the concentration of dissolved H_2_ (Kelly et al., 2019). It was also found that corn oil and fish oil supplementation lowered methyl-compounds plasma availability (Jenkins and Kramer, 1990). These findings indicated that goats were more responsive to alterations in methylotrophic pathway supplies.

A limitation of this study is the small number of animals per species, inherent to the 4 × 4 Latin-square design and the complexity of combining respiration-chamber measurements with rumen metatranscriptomics. Although repeated observations across periods increased the number of diet-specific samples, the animal remained the experimental unit. Therefore, the high-dimensional metatranscriptomic, network, clustering, and sPLS analyses should be interpreted as exploratory and hypothesis-generating. Validation in larger independent cohorts will be required to confirm the candidate microbial markers and species-specific mechanisms identified here.

This exploratory study provides valuable data explaining the differences in microbial mechanisms involved in CH_4_ production between cows and goats.

## 7 Conclusion

This study provides a direct comparison of cow and goat rumen microbiomes by integrating CH₄-emission traits with metatranscriptomic profiles under the same dietary and experimental conditions. Moreover, the rumen microbial ecosystem was only poorly characterized in goats before. Our results showed that in both ruminant species, CH_4_ yield was closely associated with genes involved in carbohydrate metabolism, highlighting the importance of diet-driven susbtrate availability for methanogenesis. Candidate microbial markers from methanogenesis-related pathways differed between cows and goats, despite a similar reduction in CH₄ emissions under the COS diet. These findings suggest that cows and goats may differ in the microbial features associated with diet-induced CH₄ mitigation. However, because the high-dimensional analyses were exploratory and microbial responses were mainly analyzed within each species, these results should be interpreted as species-associated patterns rather than definitive species-specific mechanisms. Further studies with larger cohorts and designs specifically powered to test interspecies differences are required to validate these candidate microbial markers and to determine whether rumen physiology, feeding behavior, or rumen dynamics contribute causally to microbial responses involved in CH₄ mitigation.

## Data Availability

The datasets presented in this study can be found http://www.ncbi.nlm.nih.gov/bioproject/762012. The code is available at https://github.com/kfcorraljara/Cows-and-Goats-Microbiome-Data-Analysis.

## Supplementary material

Supplementary Figure 1 Experimental design and analytical workflow. **(A)** Cows and goats were assigned to one of four dietary treatments for 28 days: control diet (CTL), control diet supplemented with corn oil and wheat starch (COS), marine algae powder (MAP), or hydrogenated palm oil (HPO). Feed intake, body weight, milk production, enteric methane (CH₄) and carbon dioxide (CO₂) emissions, and ruminal fermentation parameters were evaluated. Ruminal fluid samples collected on day 28 were used for volatile fatty acid (VFA), ammonia, protozoal, and RNA-sequencing analyses. **(B)** Workflow of the metatranscriptomic analysis. KEGG orthologs (KOs) transcript and operational taxonomic unit (OTU) abundance tables were analyzed using DESeq2 for differential abundance, CCREPE and Cytoscape for co-occurrence network construction and visualization, K-means clustering in R, KEGGREST for pathway enrichment analyses, and mixOmics for sparse Partial Least Squares (sPLS) analysis to identify variables associated with CH₄ emissions.

Supplementary Figure 2 Venn diagrams illustrating shared and unique differentially abundant features across diet comparisons. **(A)** Differentially abundant operational taxonomic units (deOTUs) in cows, **(B)** differentially expressed KEGG orthologs (deKOs) in cows, **(C)** deOTUs in goats, and **(D)** deKOs in goats identified across the three dietary comparisons: corn oil and wheat starch supplementation treatment versus control diet (COS vs. CTL), COS versus marine algae powder supplementation treatment (COS vs. MAP), and COS versus hydrogenated palm oil supplementation treatment (COS vs. HPO). Numbers inside the Venn diagrams indicate the number of deOTUs or deKOs identified in the differential analysis for each comparison or shared between comparisons.

Supplementary Figure 3 Piechart of pathway enrichment analysis in COS vs. CTL comparison in cows and goats. **(A)** Pathways related to cluster 1 in cows, **(B)** pathways related to cluster 5 in cows, **(C)** pathways related to cluster 5 in goats, **(D)** pathways related to cluster 1 in goats.

Supplementary Figure 4 Integrated network linking metabolic reactions, pathways, and identified metabolites in the COS vs. CTL comparison in cows. Blue nodes represent deKOs from cluster 1, red nodes represent deKOs from cluster 7, and yellow nodes represent deKOs from cluster 5. Green squares indicate pathway names, while white nodes represent metabolic reactions.

Supplementary Figure 5 Co-occurrence networks of differentially abundant OTUs (deOTUs), differentially expressed KEGGs (deKOs) and fermentation parameters in the COS vs. MAP comparison in cows. Red nodes represent lower abundant deKOs, green nodes are higher abundant deKOs, blue nodes are higher abundant deOTUs, pink nodes represent lower abundant deOTUs and yellow nodes are fermentation parameters and protozoa counts.

Supplementary Figure 6 Co-occurrence networks of differentially abundant OTUs (deOTUs), differentially expressed KEGGs (deKOs) and fermentation parameters in the COS vs. HPO comparison in cows. Red nodes represent lower abundant deKOs, green nodes are higher abundant deKOs, blue nodes are higher abundant deOTUs, pink nodes represent lower abundant deOTUs and yellow nodes are fermentation parameters and protozoa counts.

Supplementary Figure 7 Integrated network linking metabolic reactions, pathways, and identified metabolites in the COS vs. CTL comparison in goats. Blue nodes represent deKOs from cluster 1 and yellow nodes represent deKOs from cluster 5. Green squares indicate pathway names, while white nodes represent metabolic reactions.

Supplementary Table 1 Dietary formulations (as described in Martin et al., 2021).

Supplementary Table 2 Bioinformatic analysis metrics.

Supplementary Table 3 The proportion deOTUs specific to COS vs. CTL, COS vs. MAP and COS vs. HPO from the differential occurrence analysis in cows.

Supplementary Table 4 The proportion of deKOs specific to COS vs. CTL, COS vs. MAP and COS vs. HPO from the differential occurrence analysis in cows.

Supplementary Table 5 The proportion of deOTUs specific to COS vs. CTL, COS vs. MAP and COS vs. HPO from the differential occurrence analysis in goats.

Supplementary Table 6 The proportion of deKOs specific to COS vs. CTL, COS vs. MAP and COS vs. HPO from the differential occurrence analysis in goats.

Supplementary Table 7 Network components and clustering analysis in cows from COS vs. CTL comparison.

Supplementary Table 8 Network components and clustering analysis in cows from COS vs. MAP comparison.

Supplementary Table 9 Network components and clustering analysis in cows from COS vs. HPO comparison.

Supplementary Table 10 Network components and clustering analysis in goats. Data from COS vs. CTL comparison.

Supplementary Table 11 sPLS analysis results for (A) COS vs. CTL cluster 1 in cows; (B) COS vs. CTL cluster 5 in cows; (C) COS vs. CTL cluster 1 in goats and (D) COS vs. CTL cluster 5 in goats.
