# Supplementary material for: Species-specific rumen microbial responses to dietary inhibition of methanogenesis in cows and goats
Source: Front Microbiol. 2026 Sep 4;17:1852812. doi: 10.3389/fmicb.2026.1852812 (PMC13585762; doi:10.3389/fmicb.2026.1852812)

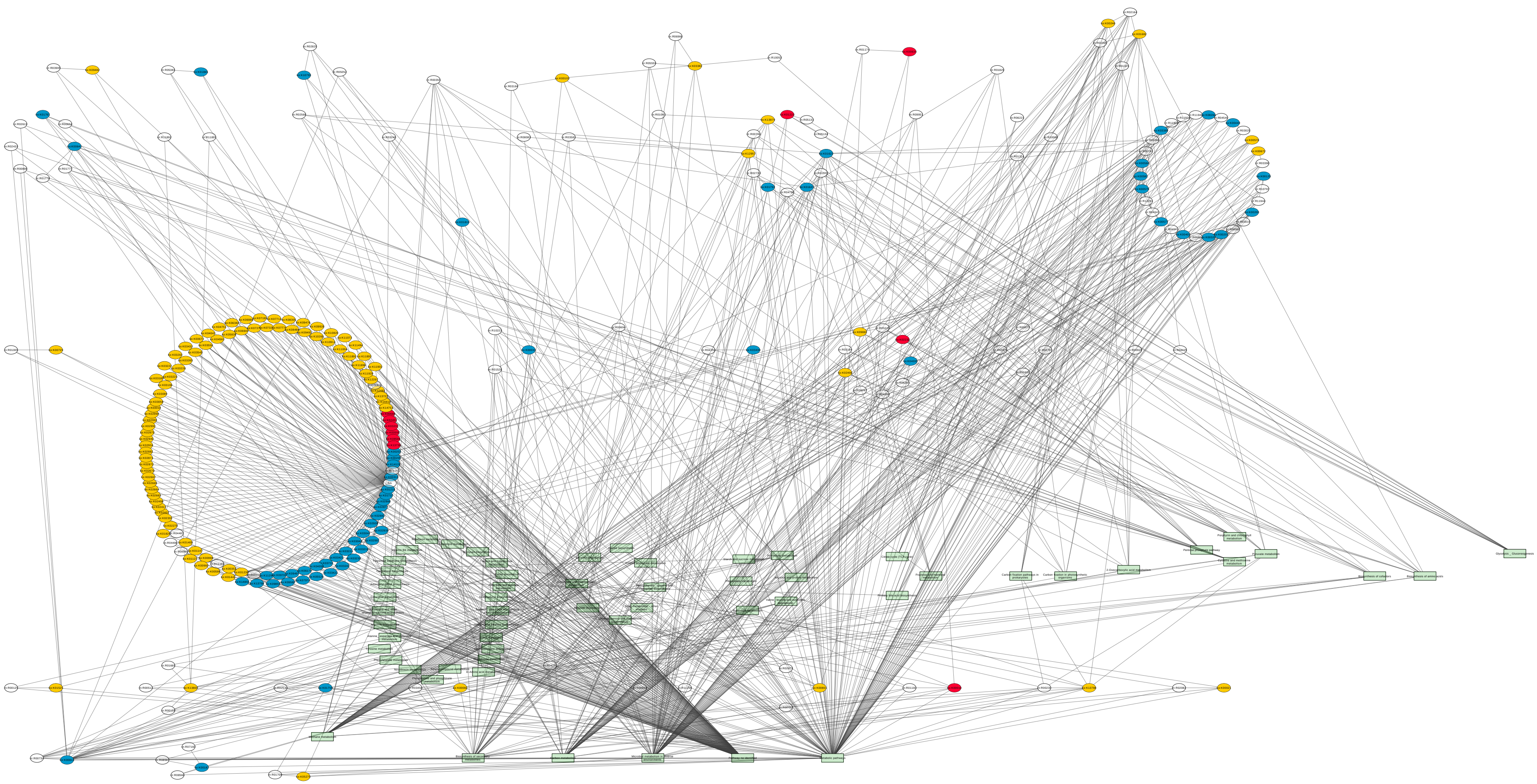

Supplement: Supplementary file 1 [file Image_4.PDF]
