# Supplementary material for: Species-specific rumen microbial responses to dietary inhibition of methanogenesis in cows and goats
Source: Front Microbiol. 2026 Sep 4;17:1852812. doi: 10.3389/fmicb.2026.1852812 (PMC13585762; doi:10.3389/fmicb.2026.1852812)
Supplement: Supplementary file 4 [file Image_7.PDF]

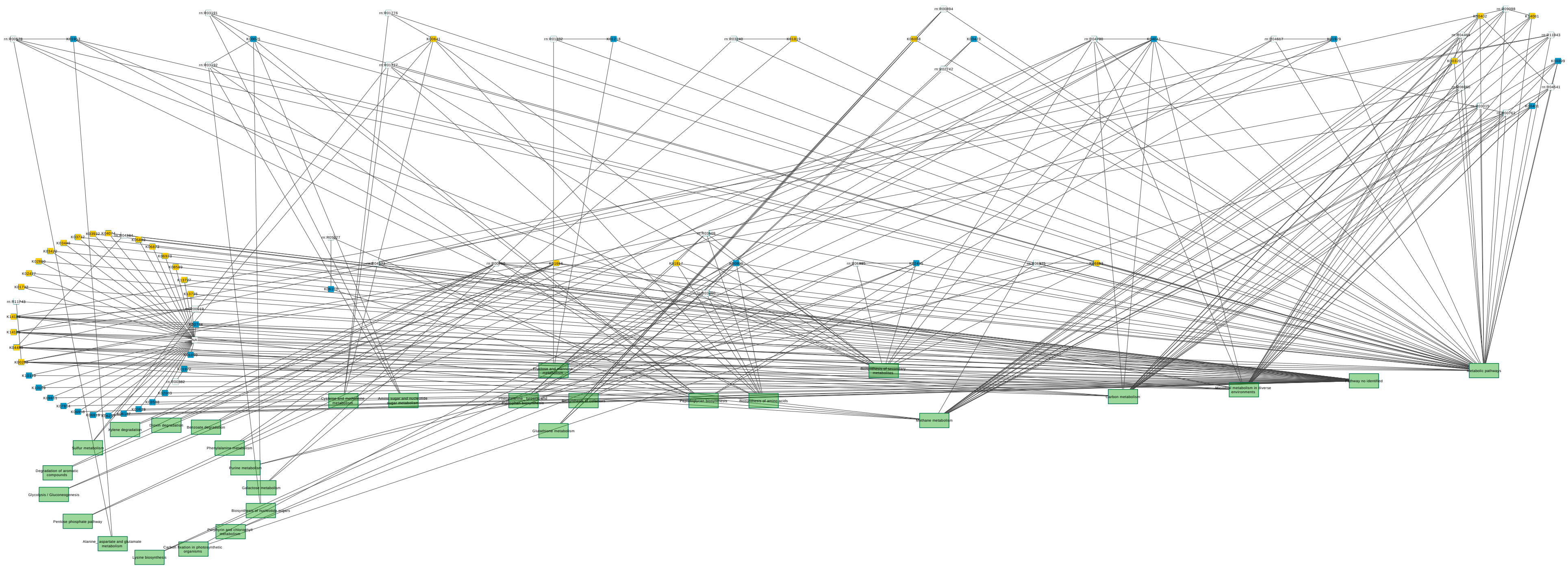
Fructose and mannose metabolism
Biosynthesis of secondary metabolites
Metabolic pathways
Pathway no identified
Microbial metabolism in diverse environments
Carbon metabolism
Methane metabolism
Cysteine and methionine metabolism
Amino sugar and nucleotide sugar metabolism
Phenylalanine, tyrosine and tryptophan biosynthesis
Biosynthesis of cofactors
Peptidoglycan biosynthesis
Biosynthesis of amino acids
Glutathione metabolism
Xylene degradation
Dioxin degradation
Benzoate degradation
Sulfur metabolism
Phenylalanine metabolism
Degradation of aromatic compounds
Purine metabolism
Glycolysis / Gluconeogenesis
Galactose metabolism
Biosynthesis of nucleotide sugars
Pentose phosphate pathway
Porphyrin and chlorophyll metabolism
Alanine, aspartate and glutamate metabolism
Carbon fixation in photosynthetic organisms
Lysine biosynthesis
